# Short-term effects of calcium ions on the apoptosis and onset of mineralization of human dental pulp cells *in vitro* and *in vivo*

**DOI:** 10.3892/ijmm.2015.2218

**Published:** 2015-05-22

**Authors:** SHAOFENG AN, YAN GAO, YIHUA HUANG, XIAOQIONG JIANG, KE MA, JUNQI LING

**Affiliations:** Department of Operative Dentistry and Endodontics, Guanghua School of Stomatology, Hospital of Stomatology, Sun Yat-sen University; Guangdong Provincial Key Laboratory of Stomatology, Guangzhou, Guangdong 510055, P.R. China

**Keywords:** human dental pulp cells, pulp capping, calcium, mineralization, apoptosis

## Abstract

Calcium ions (Ca^2+^) are a major constituent of most pulp-capping materials and have an important role in the mineralization of human dental pulp cells (hDPCs). A previous study by our group has shown that increased levels of Ca^2+^ can promote hDPC-mediated mineralization in long-term cultures (21 days). However, the initiation of mineralization occurs in the early stage of osteogenic inductive culture, and the effects of Ca^2+^ on the mineralization of hDPCs in short-term cultures (five days) have not been studied in detail. Furthermore, the underlying mechanism by which Ca^2+^ stimulates the mineralization of hDPCs has remained controversial. A strong correlation between mineralization and cell apoptosis and/or death has been identified. Thus, the present study hypothesized that Ca^2+^ may promote the onset of hDPC-mediated mineralization through inducing their apoptosis and/or death. To verify this hypothesis, Ca^2+^ was added to the growth culture medium and osteogenic culture medium at various concentrations. Alizarin Red S staining and reverse transcription-polymerase chain reaction analysis were used to evaluate the onset of mineralization. Furthermore, the cell counting kit-8 and fluorescein isothiocyanate-Annexin V/propidium iodide double-staining method were adopted to detect the proliferation and apoptosis of hDPCs in the growth culture medium. An animal experiment and scanning electron microscopic observation of ceramic graft implants were applied to measure the mineralization *in vivo*. The results showed that 5.4 and 9.0 mM Ca^2+^ accelerated the onset of mineralized matrix nodule formation, promoted osteopontin mRNA expression and induced marked cell apoptosis and necrosis, but had no obvious effect on cell proliferation. These findings indicated a positive association between cell apoptosis and/or death and the timing of formation as well as the quantity of extracellular mineralization induced by Ca^2+^ in short-term cultured hDPCs.

## Introduction

The main goal of restorative dentistry is to re-construct the tooth and maintain the vitality and function of human dental pulp tissue (1). Numerous dental clinical techniques, including the removal of dental caries and tooth preparation, may lead to accidental dental pulp exposure (1,2). Dental pulp tissue has been shown to harbor various populations of multipotent stem/progenitor cells. The main type of cell linage is that of human dental pulp cells (hDPCs), which have multipotent differentiation characteristics and are therefore regarded as potential sources for the re-generation of dentin under certain circumstances (3). For this reason, in permanent teeth with normal pulp or those diagnosed with reversible pulpitis, direct pulp capping is a feasible therapeutic method to preserve the vitality of an exposed and potentially infected pulp (4). In this treatment, once the exposure occurred, the tooth must be isolated from the saliva to prevent contamination, and then an appropriate pulp-capping material is placed directly over the exposed pulp to induce tertiary dentin formation (4,5).

Although there are various factors affecting the treatment outcome of direct pulp capping, control of infection and biocompatibility of the capping materials are considered as pivotal factors in influencing treatment outcome (6,7). Over the last few decades, different formulations of calcium hydroxide [Ca(OH)_2_] and its derivatives have been used for direct pulp capping therapies (8). Despite its wide use, Ca(OH)_2_ is not ideally compatible with pulp capping. It has three major drawbacks: The porosity of its induced dentinal bridge; inferior adherence to the dentin; and the microleakage produced from its decomposition (9). Therefore, to achieve good clinical results, numerous clinicians and researchers have performed studies with the aim of improving the properties of pulp-capping materials (5). In recent years, several novel biomaterials, including β-tri-calcium phosphate ceramics (β-TCP), calcium silicate cements and mineral trioxide aggregates (MTA) have been recommend as alternatives to Ca(OH)_2_-based pulp-capping materials (10,11).

Calcium ions (Ca^2+^) are major components of the pulp-capping materials currently used in clinical practice (12). Ca^2+^ released from the pulp-capping materials reacts with carbonates in the pulp tissue, forming calcium carbonate, which affects hDPC proliferation and differentiation, and thus contributes to the onset of mineralization (12–14). A previous study by our group has shown that increased levels of Ca^2+^ can promote the mineralization of hDPCs in long-term cultures (21 days) (15). However, the trigger for mineralization occurs in the early stages of osteogenic inductive culture, and the effects of Ca^2+^ on the mineralization of hDPCs in short-term cultures have not been studied in detail. Furthermore, the underlying mechanisms by which Ca^2+^ stimulates the mineralization of hDPCs have remained controversial. Previous studies have indicated that there is a strong correlation between mineralization and cell apoptosis and/or death (16). Thus, the present study hypothesized that Ca^2+^ may promote the onset of mineralization in hDPCs through inducing their apoptosis in the early stage of differentiation. Therefore, the objective of the present study was to assess this hypothesis through investigating the short-term effects of Ca^2+^ on the apoptosis and mineralization of hDPCs. The culture media was supplemented with various concentrations of Ca^2+^. hDPCs were short-term cultured in the osteogenic media (five days) to investigate cell mineralization, and in growth media to examine cell apoptosis and proliferation. Finally, an animal experiment was performed to test the performance of hDPCs on porous three-dimensional scaffolds cultured in the growth media in the presence of Ca^2+^.

## Materials and methods

### Isolation and expansion of hDPCs

hDPCs were isolated from healthy impacted teeth extracted from five subjects <30 years of age, and informed consent was acquired from all subjects. Primary cultures of hDPCs were established as previously described using an explant culture method (15). All experimental protocols used were approved by the Ethics Committee of Guanghua School of Stomatology, Hospital of Stomatology, Sun Yat-sen University (Guangzhou, China). α-modified Minimum Essential Medium (α-MEM/High Glucose; HyClone, Beijing, China) was supplemented with 10% fetal bovine serum (FBS; Biological Industries, Kibbutz Beit Haemek, Israel) and 2% (v/v) penicillin/streptomycin (Invitrogen Life Technologies, Carlsbad, CA, USA), and used for primary culture of hDPCs. The method for culturing the hDPCs was as previously described (15). The cells were sub-cultured at a 3:1 ratio after reaching ~80% confluency through trypsinization (trypsin/EDTA; Invitrogen Life Technologies). Cells at the fifth passage were used in the subsequent experiments.

### Ca^2+^ supplementation

Ca^2+^ supplementation was performed using CaCl_2_ as a sterile 1.8 M solution in double distilled water. The Ca^2+^ concentration in commercial α-MEM media is 1.8 mM. Based on this concentration in the culture medium, which was used as the control, various quantities of Ca^2+^ were added immediately prior to incubation of the cell cultures to achieve final concentrations of 5.4, 9.0, 12.6 and 16.2 mM. Accordingly, treatment groups are being referred to as Ca1.8, Ca5.4, Ca9.0, Ca12.6 and Ca16.2. To assess cell mineralization and apoptosis, Ca^2+^ was also added to the osteogenic differentiation medium [α-MEM with 10% FBS, 2% penicillin/streptomycin (Invitrogen Life Technologies), 10^−8^ M dexamethasone, 50 mg/ml ascorbic acid, and 8 mM β-glycerophosphate (all from Sigma-Aldrich, Gillingham, Dorset, UK)] and basic growth medium [α-MEM containing 10% FBS (Invitrogen Life Technologies) and 2% penicillin/streptomycin].

### Cellular mineralization

To observe the effect of Ca^2+^ levels on cell mineralization, mineralized nodules were detected using an Alizarin Red S staining kit (Genmed, Shanghai, China) according to the manufacturer’s instruction at days 3 and 5 of osteogenic inductive culture. The concentrations of Ca^2+^ tested in were as follows: 1.8 mM Ca^2+^(Ca1.8; control), Ca5.4, Ca9.0, Ca12.6 and Ca16.2 mM. hDPCs were cultured at a density of 1×10^4^/cm^2^ in 24-well microplates. A series of cell-free controls parallel to each group tested were used to check whether spontaneous precipitation occurred following Ca^2+^ supplementation under identical conditions to those in the cell culture. The stained cells in each well were observed using an inverted phase-contrast microscope (Olympus IX41; Olympus, Tokyo, Japan), and microscopic images were captured at different magnification.

### RNA extraction and reverse transcription-polymerase chain reaction (RT-PCR)

Based on the results of the cell mineralization assay, 5.4 and 9.0 mM Ca^2+^ were defined as the experimental conditions for the subsequent assays. It is known that osteopontin (OPN) is required for the initiation of hard-tissue mineralization (16). Hence, after five days of osteogenic induction culture, the gene expression levels of OPN were monitored by RT-PCR as described previously (17). The total RNA of hDPCs from the 5.4- and 9.0 mM Ca^2+^ groups was extracted using TRIzol reagent (Invitrogen Life Technologies), and reverse transcribed by RevertAid™ Moloney Murine Leukemia Virus Reverse Transcriptase (MBI Fermentas, Thermo Fisher Scientific, Waltham, MA, USA). Target gene expression was standardized relative to the housekeeping gene GAPDH. The 30 *µ*l PCR mixture consisted of 3 *µ*l 10X Taq Buffer with (NH_4_)_2_SO_4_, 3 *µ*l 2 mM dNTP Mix, 2 *µ*l 25 mM MgCl_2_, 1.5 U Taq DNA Polymerase, 0.6 *µ*l (10 pg-1 *µ*g) cDNA template, and 10 *µ*M of each primer set. The reaction conditions for the PCR were as follows: 94°C (3 min), followed by 28 cycles of 94°C (30 sec), 60°C (30 sec) and 72°C (20 sec), with a final 3-min extension at 72°C. Forward and reverse primers were as follows: forward, GCCGAGGTGATAGTGTGGTT and reverse, TGAGGTGATGTCCTCGTCTG, with a product size of 101 bp. The primer sequence for GAPDH was as follows: forward, CATGTTCCAATATGATTCCACC; and reverse, GATGGGATTTCCATTGATGAC, with a product size of 88 bp. All primers were custom made by Invitrogen Life Technologies. The PCR product was separated with DYY-12 electrophoresis apparatus (Beijing Liuyi Instrument Factory, Beijing, China) by 2% agarose at 120V.

### Cell viability assay

The cell proliferation was measured using the Cell Counting kit-8 (CCK-8; Beyotime Institute of Biotechnology, Shanghai, China) in growth media containing the abovementioned concentrations of Ca^2+^. Briefly, cells were seeded into the 96-well plate at a density of 2×10^3^ cells/well, incubated for 24 h and starved in serum-free media for another 24 h. Subsequently, media in every well were replaced with the designated culture medium. After 1, 3, 5 and 7 days of culture, the medium was replaced with 15 *µ*l CCK-8 solution. This solution was incubated with the cell cultures at 37°C for 4 h in a humidified atmosphere containing 5% CO_2_. Optical densities of the supernatants were measured at 490 nm with an ELISA spectrophotometer (Infinite 200; Tecan, Männedorf, Switzerland).

### Determination of cell apoptosis

hDPCs were seeded into six-well culture plates in the basic growth medium for 24 and 72 h, and 1×10^5^ cells were harvested with 0.25% trypsin/EDTA (Invitrogen Life Technologies) and re-suspended in binding buffer (BD Biosciences, Franklin Lakes, NJ, USA). Apoptotic cells were identified by double supravital staining using the recombinant fluorescein isothiocyanate-conjugated Annexin-V and propidium iodide (Annexin V/PI) Apoptosis Detection kit (BD Biosciences) according to the manufacturer’s instructions. Apoptotic cell death was examined by FACSCalibur flow cytometry (BD Biosciences). Viable cells were Annexin V^−^/PI^−^, early apoptotic cells were Annexin-V^+^/PI^−^, late apoptotic cells were Annexin-V^+^/PI^+^ and necrotic cells were Annexin V^−^/PI^+^.

### Animal experiment

Based on the results of cell mineralization and apoptosis, the 5.4 mM Ca^2+^ group was selected for *in vivo* analysis. Six-week-old female severe combined immu nodeficient (SCID) mice (weighing 25 g; Laboratory Animal Center, Sun Yat-sen University, Guangzhou, China) were used for the subcutaneous transplantation study. Animal protocols were approved by the Sun Yat-sen University Animal Care and Use Committee (Guangzhou, China). In brief, 5×5×5-mm cube sizes of HA/TCP scaffolds were sterilized for 15 min (121°C, 15 bar pressure) by autoclaving, and soaked in DMEM supplemented with 10% FBS and 2% penicillin/streptomycin at 37°C for 4 h. For a single transplant, 20 *µ*l hDPC suspension at a density of 1×10^5^ cells/ml was seeded using a pipettor (Eppendorf Research plus; Eppendorf, Hamburg, Germany) onto hydroxyapatite/tri-calcium phosphate (HA//TCP) scaffolds (National Engineering Research Centers for Biomaterials, Chengdu, China). After incubation in the growth culture medium containing 5.4 mM Ca^2+^ for three days, the mice were anaesthetised by an intraperitoneal injection of 4% chloral hydrate (Sigma-Aldrich, St. Louis, MO, USA) (400 mg/kg dose), and then the hDPC-scaffold constructs were implanted into the dorsal surface of subcutaneous pockets. The constructs cultured in the classic α-MEM media served as the control, and HA/TCP only (without any cells) was implanted in the same animal as a blank control. Four transplants were placed into each animal. The incisions were closed with surgical staples. The mice were kept in a specific pathogen-free (SPF) environment at a constant temperature of 25±2°C with a constant humidity of 45–50%, and were fed inside the laminar air flow rack. After 1 week, the mice were sacrificed by an excessive injection of 4% chloral hydrate (Sigma-Aldrich, St. Louis, MO, USA) and implants were harvested, fixed for 1 day in 4% formaldehyde in phosphate-buffered saline, and the cell growth and mineralization were examined by scanning electron microscopy (SEM; Quanta 200; FEI, Hillsboro, OR, USA).

### Statistical analysis

Values are expressed as the mean ± standard deviation. Statistical analyses were performed by one-way analysis of variance with the least significant difference test using the SPSS 17.0 software package (SPSS, Inc., Chicago, IL, USA). P<0.05 was considered to indicate a statistically significant difference between values.

## Results

### Ca^2+^ promotes and accelerates the onset of mineralization of hDPCs

**Figure 1:**
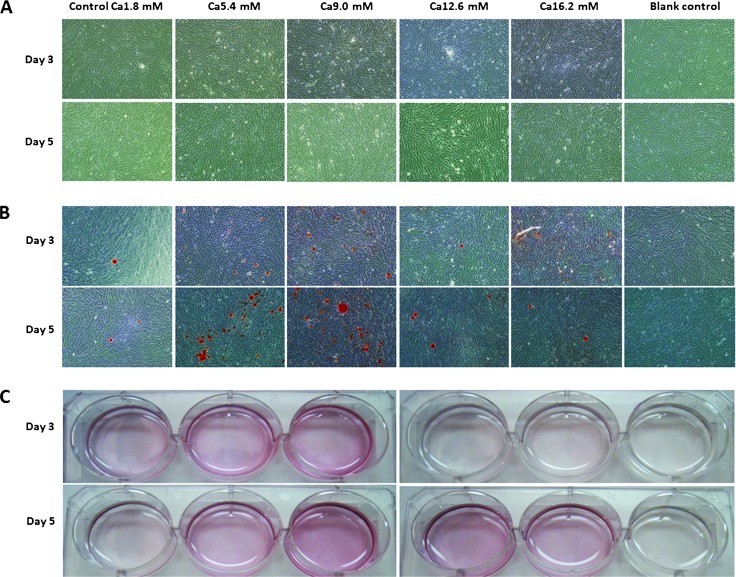
Effect of calcium ions (Ca^2+^) on the onset of mineralization in human dental pulp cells at day 3 and 5. (A) Representative micrographs prior to staining. (B) Images of Alizarin Red S staining. Obvious calcium deposition was observed in the groups treated with 5.4 and 9.0 mM Ca^2+^ (Original magnification, ×100). (C) General observation of culture wells stained with Alizarin Red S.

After three days of osteogenic differentiation culture, mineral matrix nodules first appeared in the cell cultures in the 5.4 and 9.0 mM Ca^2+^ groups, while few nodules were observed in the 12.6 and 16.2 mM Ca^2+^ groups. After five days of osteogenic inductive culture, nodules appeared in all groups; the most obvious mineralization nodes were found in the 5.4 and 9.0 mM Ca^2+^groups. Fig. 1A shows images prior to staining, Fig. 1B shows images of Alizarin Red S staining of mineralization in hDPCs and Fig. 1C shows Alizarin Red S-stained culture wells.

### Ca^2+^ enhances the expression of OPN

**Figure 2:**
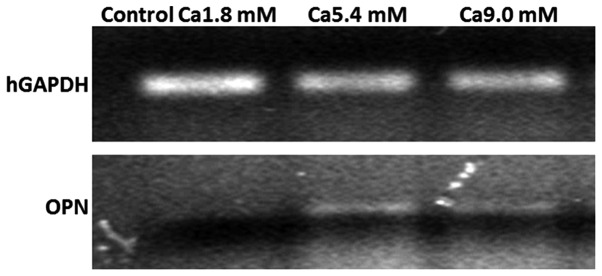
Reverse transcription quantitative polymerase chain reaction analysis of OPN gene expression. A representative agarose gel is shown. OPN was not expressed in the control group, while OPN mRNA levels were upregulated in the Ca^2+^-treated groups. OPN, osteopontin.

OPN gene expression was negative in the control group, while hDPCs cultured in media with enhanced Ca^2+^ levels displayed mRNA expression of OPN. Amongst all groups, 5.4 mM Ca^2+^ caused the most marked increase in OPN mRNA expression (Fig. 2).

### Ca^2+^ does not affect the proliferation of hDPCs

**Figure 3:**
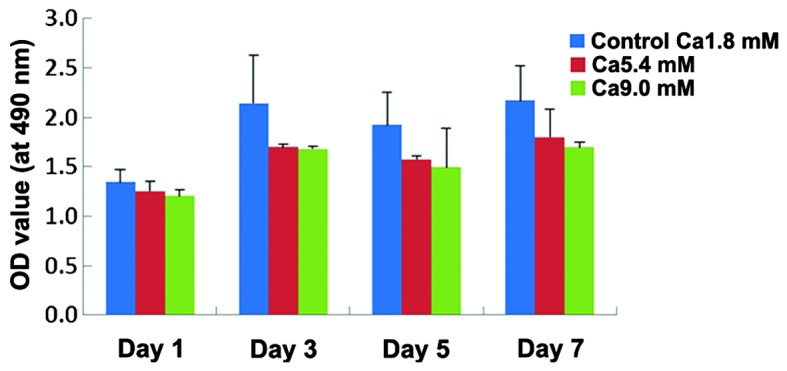
Effect of calcium ions on the proliferation of hDPCs. Increased levels of Ca^2+^ decreased the proliferation of the hDPCs in all experimental groups; however, there were no significant differences between the experimental groups and the control group (P>0.05). Values are expressed as the mean ± standard deviation. hDPCs, human dental pulp cells; OD, optical density.

As compared with that in the control group (Ca1.8 mM), the cell number of the hDPCs in all experimental groups analyzed was decreased at all time-points. However, there were no statistically significant differences in cell proliferation levels between these experimental groups and the control (P>0.05) (Fig. 3).

### Ca^2+^ induces apoptosis and necrosis of hDPCs

**Figure 4:**
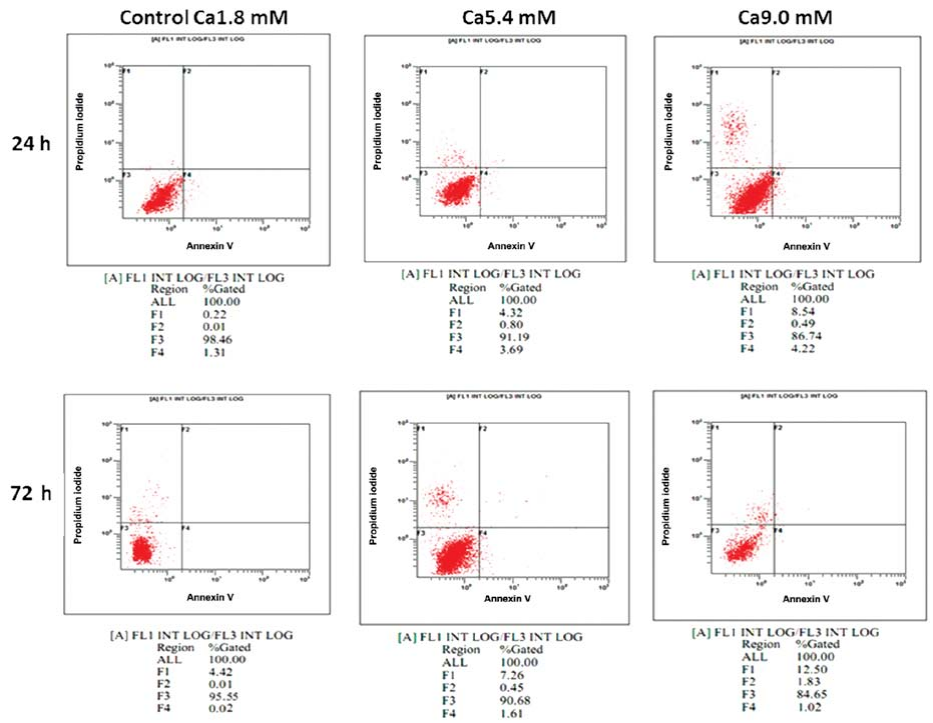
Detection of cell apoptosis. Representative scatter photos are shown. Apoptosis was quantified by fluorescence-assisted cell sorting analysis after staining with Annexin V and PI at 24 or 72 h. Viable cells were Annexin V^−^/PI^−^ (F3), early apoptotic cells were Annexin V^+^/PI^−^ (F4), late apoptotic cells were Annexin V^+^/PI^+^ (F2) and necrotic cells were Annexin V^−^/PI^+^ (F1). The cells treated with higher concentrations of calcium ions showed higher apoptotic and necrotic rates compared with those in the control group. PI, propidium iodide.

After incubation in media supplemented with 5.4 and 9.0 mM Ca^2+^ for 24 or 72 h, hDPCs exhibited higher apoptotic and necrotic rates compared with those in the control group. As compared with the control group, following incubation with 9.0 mM Ca^2+^ for 24 h, the early apoptotic rate was increased (1.31 vs. 4.22%) and the necrotic rate was elevated (0.22 vs. 8.54%). Furthermore, as compared with the control group, following incubation with 9.0 mM Ca^2+^ for 72 h, the early apoptotic rate was increased (0.02 vs. 1.02%) and the necrotic rate was elevated (4.42 vs. 12.50%) (Fig. 4).

### Ca^2+^ inhibits hDPC growth and induces their mineralizaion in vivo

**Figure 5:**
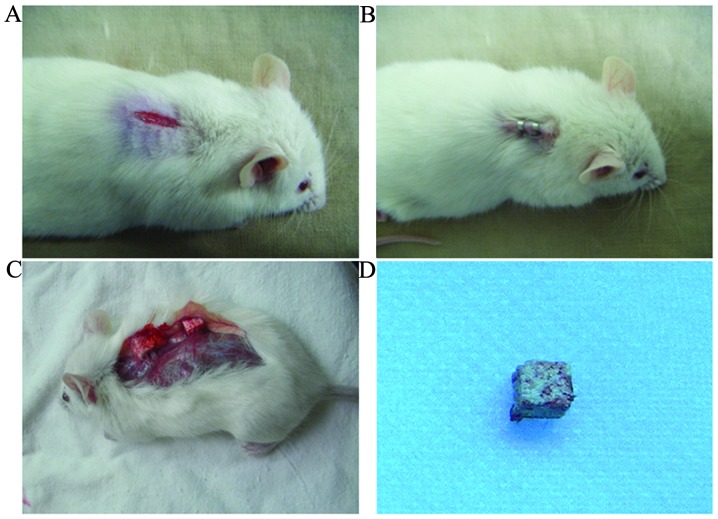
Procedures of the animal study. Six-week-old female SCID mice were used for the subcutaneous transplantation study. (A) Midlongitudinal skin incisions of ~1 cm in length were made on the dorsal surface of each mouse, and subcutaneous pockets were generated by blunt dissection. (B) Each animal was implanted with four three-dimensional porous ceramic graft transplants containing human dental pulp cells incubated with 1.8 or 5.4 mM Ca^2+^, with one single transplant per pocket. The incisions were closed with surgical staples. (C and D) Implants were harvested at day 7 after transplantation, fixed for 1 day in 4% formaldehyde in phosphate-buffered saline, and the cell growth and mineralization were examined by scanning electron microscopy.

**Figure 6:**
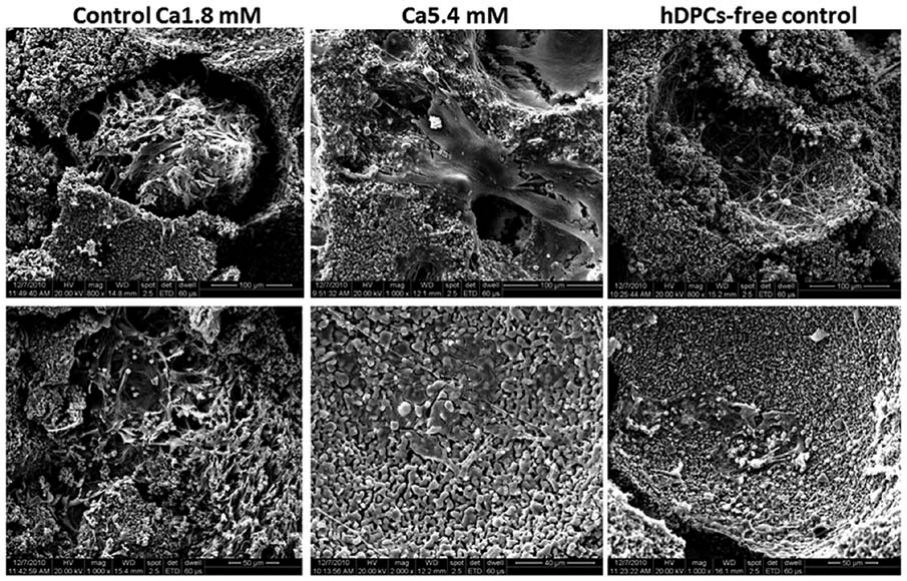
Scanning electron microscopy images of hDPC-HA/TCP complexes harvested from SCID mice (scale bar: Top row, 100 *µ*m; bottom row, 50 *µ*m). In the control Ca1.8 mM HA/TCP scaffolds, a large number of hDPCs proliferated significantly and aggregated to form a dense multilayer with clumping. By contrast, hDPCs cultured in medium containing 5.4 mM Ca^2+^ displayed undefined cell borders and intercellular fusion, and numerous extracellular matrixes on the scaffolds’ surface were formed. hDPC; human dental pulp cell; HA/TCP, hydroxyapatite/tri-calcium phosphate.

At 7 days following implantation of HA/TCP scaffolds containing hDPCs incubated with 5.4 mM Ca^2+^ into SCID mice (Fig. 5), the transplants were analyzed using SEM. In the control group, a large number of hDPCs successfully adhered to the HA/TCP scaffolds and had proliferated, resulting in a dense multilayer and spread with clumping of the cells into the three-dimensional network of the scaffold. However, the scaffolds containing hDPCs cultured in medium supplemented with 5.4 mM Ca^2+^ displayed intercellular fusions and produced an extracellular matrix on the surface (Fig. 6).

## Discussion

Mineralization is an essential requirement for normal bone and tooth development, which is generally achieved through the function of two cell types, osteoblasts and chondrocytes (18). It is generally thought that tissue mineralization is achieved under appropriate conditions, which are generated through the interaction of three key elements: The extracellular matrix, extracellular levels of calcium and inorganic phosphate ions, and mineralization inhibitors that may be expressed systemically or locally (19,20).

In the present study, 5.4 and 9.0 mM Ca^2+^ promoted the mineralization of hDPCs at day 3, but 12.6 and 16.2 mM Ca^2+^ did not stimulate the mineralization. This result was similar to that of a study by Maeno *et al* (21), who reported similar effects of Ca^2+^, which significantly influenced the mineralization of osteoblasts at levels of 6–10 mM, whereas further increases beyond this range led to a decrease in the amount of mineral deposition. These findings suggested that there is no linear trend between the onset of mineralization and extracellular Ca^2+^ concentrations, and there appears to be a limit beyond which increases in the Ca^2+^ concentration do not lead to continued increases in hDPCs mineralization. This marked decrease in mineralization in the 12.6 and 16.2 mM Ca^2+^ groups is most likely a result of the cytotoxic effects outweighing any benefit in mineral production. The mineralization assay of the present study also showed that 5.4 and 9.0 mM Ca^2+^ induced the maximum amount of mineral nodules at days 3 and 5. Based on this result and that of a previous study by our group (15), it can be concluded that an optimal extracellular concentration of Ca^2+^ is an essential factor to trigger and maintain the hDPCs-mediated mineralization process.

OPN is a key marker of osteogenic differentiation and mineralization. High levels of OPN are associated with the initiation of tissue mineralization and ectopic calcification (22). However, the exact function of OPN in mineralization has remained elusive. It is now accepted that the presence of high levels of OPN in calcified soft tissues is an effect of calcification, rather than its cause (22,23). Is is though that OPN functions in the prevention of crystal formation in the mineralization process (24). The results of the RT-PCR analysis of the present study revealed an obvious increase of OPN mRNA in response to elevated extracellular Ca^2+^, indicating that the elevated calcium levels triggered hDPCs-mediated mineralization. By contrast, OPN mRNA was not detected in the control group. These findings suggested a positive association between the onset of hDPCs-mediated mineralization and OPN gene expression.

The biochemical mechanisms that initiate mineralization subsequent to the increase in the Ca^2+^ concentration have remained controversial. A marked correlation between mineralization and cell death has been observed, and in particular, pathological mineralization has often been associated with apoptotic or necrotic processes (25). To investigate the correlation between the mineralization induced by elevated levels of Ca^2+^ and cell apoptosis, the present study determined the apoptotic and necrotic rates of hDPCs cultured in growth medium supplemented with 5.4 and 9.0 mM Ca^2+^ using Annexin V/PI staining followed by flow cytometric analysis. The results showed that elevated concentrations of Ca^2+^ increased the apoptotic rate, and the number of necrotic cells was also significantly increased. Hence, it was speculated that the mineralization of hDPCs induced by Ca^2+^ is associated with the induction of cell apoptosis or cell death.

Finally, SEM images of hDPCs engrafted onto HA/TCP scaffolds and incubated in SCID mice for 7 days showed differences in morphology between the hDPCs cultured in the presence of 5.4 mM Ca^2+^ and the control cells. The hDPCs cultured in medium containing 5.4 mM Ca^2+^ displayed reduced proliferation compared with that of the control cells and had produced a larger amount of extracellular matrix. Apart from the effect of Ca^2+^ in the culture media, free Ca^2+^ released from the scaffolds is also an important factor that contributes to the mineralization (15,26–28). The results of the present study indicated that 5.4 mM Ca^2+^ promoted hDPC-mediated mineralization and contributed to the formation of a calcific barrier.

In conclusion, the results of the present study and those of previous studies indicated the significance of optimal extracellular Ca^2+^ levels in stimulating and maintaining hDPCs-mediated mineralization. However, mineralization induced by elevated concentrations of Ca^2+^ was likely to be a trigger of apoptosis and/or necrosis of hDPCs. In conclusion, the present study provided a rationale for the design of novel pulp-capping materials, suggesting that an optimal extracellular Ca^2+^ concentration is able to improve clinical outcomes.
